# Pilot wrap-around support initiative: Enhanced support for internally funded CTSA pilot awards

**DOI:** 10.1017/cts.2025.10071

**Published:** 2025-06-09

**Authors:** Kelsey C. Stoltzfus, Tyler J. Deal, John P. Vanden Heuvel, Aron E. Lukacher, Susan Simkins, Nikolay V. Dokholyan, Jennifer L. Kraschnewski

**Affiliations:** 1 Penn State Clinical and Translational Science Institute, Penn State College of Medicine, Hershey, PA, USA; 2 Department of Molecular Toxicology, Penn State College of Agricultural Sciences, University Park, PA, USA; 3 Department of Cell and Biological Systems, Penn State College of Medicine, Hershey, PA, USA; 4 Department of Psychology, Penn State College of the Liberal Arts, University Park, PA, USA; 5 Department of Neuroscience & Experimental Therapeutics, Penn State College of Medicine, Hershey, PA, USA; 6 Department of Medicine, Pediatrics, and Public Health Sciences, Penn State College of Medicine, Hershey, PA, USA

**Keywords:** Pilot funding, clinical and translational science award, research support, research infrastructure, team science

## Abstract

The Penn State Clinical and Translational Science Institute (CTSI) Pilot Wrap-Around Support Initiative, implemented in the 2023 pilot funding cycle, was designed to help research teams access comprehensive research support for their pilot projects throughout the entire funding cycle. The overall goal of the initiative is to support researchers to improve timely and successful completion of project aims with a high likelihood of receiving follow-on external funding to advance clinical and translational science. This initiative builds upon existing CTSI resources by adding new support mechanisms, including Core consultations during application development, a pilot project Principal Investigator (PI) orientation meeting, and team science collaboration planning for funded teams. In the 2023 and 2024 funding cycles, we received 55 letters of intent and 23 invited full applications. Pilot project PIs engaged in each of our new and existing support mechanisms to various degrees, resulting in six funded projects that started July 1, 2024 and four funded projects set to begin July 1, 2025. Plans for the initiative include supporting access to clinical research staff through a CTSI staffing program and overarching pilot project evaluation to guide future programmatic improvement.

## Introduction

The Penn State Clinical and Translational Science Institute (CTSI), established in 2007, supports internally funded pilot projects through an annual program called Bridges to Translation [1]. The goal of the Bridges to Translation program is to support pilot projects that build interdisciplinary connections across the translational spectrum of health and biomedical sciences. This funding mechanism seeks to fund pilot projects aimed at building linkages and overcoming roadblocks at any stage in the translational process. In addition to Bridges to Translation, in 2024, the CTSI launched a new funding mechanism called the Translational Science Pilot Funding Program. This funding mechanism seeks to fund pilot projects aimed at generating scientific and operational innovations to overcome longstanding barriers to translational research, with a focus on translational science. Additionally, each funding mechanism gives special consideration to projects that align with our CTSI mission and vision focused on rural health. Since 2012, CTSI has funded 138 pilot projects. The CTSI pilot program has been successful in supporting externally funded research, generating over $15 of follow-on funding for every $1 invested.

Clinical research pilot studies aim to test methods and procedures of a research project on a smaller scale before expanding to a larger study population [2]. This overarching goal can be divided into smaller project aims of assessing feasibility, testing randomization and blinding processes, evaluating recruitment and consent procedures, and determining the acceptability of the proposed intervention [3]. One overall objective of supporting pilot awards is for Principal Investigators (PIs) to successfully complete these project aims as outlined in their proposal. The rates of success for pilot awards, as determined by the completion of project aims, vary across organizations based on the nature of the funding mechanism (i.e., mechanisms aimed at funding high risk, high reward projects are likely to have higher failure rates as the project is inherently riskier and more challenging). Additionally, pilot project PIs face many barriers during their project period that prevent them from successfully achieving their project aims. These may include project-specific barriers, such as difficulty recruiting the required number of study participants, or clinical and translational science barriers, such as investigator difficulty identifying available resources and services to support their research [4]. Thus, there is a need to provide additional support to PIs for both project- and institution-level challenges experienced throughout their pilot project to ensure successful project completion.

A second objective of pilot awards is for the pilot project PI to leverage the data to obtain follow-on funding from external sources, such as the National Institutes of Health (NIH) and the National Science Foundation (NSF). The process of securing additional funding is extremely competitive; only the strongest proposals with high-quality preliminary data have the greatest likelihood of success. The NIH success rate for R01 level or similar research project grant applications was 21% for fiscal year 2023 [5]. The NSF funding rate was 29% in fiscal year 2023 [6]. Because of the competitive nature of external funding, pilot project PIs receive support from their academic institution to ensure the projects are successful and produce compelling data for follow-on funding opportunities. At Penn State University, academic colleges and their departments offer varying levels of assistance to faculty preparing to submit a proposal for follow-on funding. This allows our CTSI, as a University-wide Institute and funder, to provide additional support to our pilot applicants and project awardees to ensure they are positioned for success throughout the process. Further assistance is hypothesized to yield several significant benefits: 1) stronger applications to our pilot funding mechanisms; 2) projects and scientists who successfully achieve project aims; 3) projects with a high likelihood of achieving follow-on funding; and 4) a larger return on investment for our CTSI, which includes our overall programmatic impact measured using the Translational Science Benefits Model [7].

**Figure 1.**
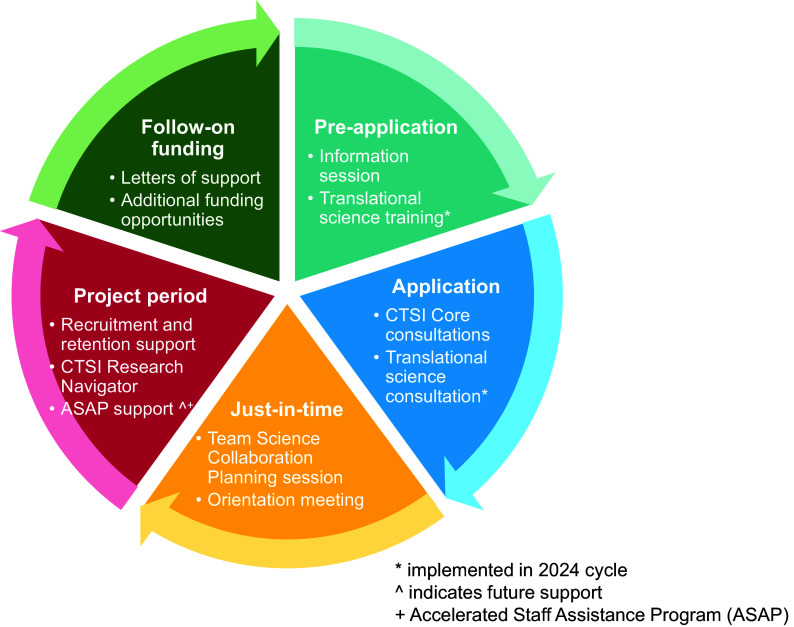
Penn state CTSI pilot wrap-around support initiative new, existing, and future support mechanisms by application stage. *implemented in 2024 cycle. ^indicates future support. +Accelerated staff assistance program (ASAP).

Launched in the summer of 2023, the Penn State CTSI Pilot Wrap-Around Support Initiative was designed to help research teams access comprehensive support for their CTSI-funded pilot projects. The resources provided through this initiative add to existing CTSI supports with new resources that we continue to develop for this initiative (Figure 1). We outline the established support mechanisms, the support mechanisms newly implemented as part of our 2023 and 2024 pilot funding cycles, and the proposed future directions of the initiative. Our primary objective in formally launching this initiative is to support applicants during the pre-application, application, and funded project periods of the funding cycle. Support mechanisms previously offered focused mainly on the project period only, while future plans for the initiative focus on enhancing the support provided during the pre-application and follow-on funding stages of the cycle. The overall goal of the Pilot Wrap-Around Support Initiative is to provide support to researchers to improve the timely and successful completion of project aims with a high likelihood of being awarded follow-on funding to advance clinical and translational science.

## Methods

A key component of programmatic success was the establishment of a new staff role, Director of Pilot Programs, to provide dedicated time and effort to enhance the support provided to our pilot funding applicants and awardees. The role of the Director is to facilitate the pilot funding process from start to finish, including organizing the final funding announcement and hosting information sessions for applicants, connecting applicants to CTSI Cores for consultative services, managing the proposal review process, and issuing award letters. Additional interaction with the pilot project PIs occurs during the just-in-time period of the award, where the Director guides awardees through institutional review board (IRB) and/or institutional animal care and use committee (IACUC) approvals and the National Center for Advancing Translational Sciences (NCATS) prior approval process required for human subjects’ research and vertebrate animal studies. Finally, the Director serves as a point of contact for awardees throughout their project period, reviewing interim progress reports and monthly financial statements to monitor the progress of the project and identify barriers and potential solutions. When questions arise, the Director provides support to the pilot project PIs or connects them to other CTSI and Penn State resources as applicable.

The Director is supported by other key members of the CTSI who contribute to the success of the initiative creation and implementation. The Translational Endeavors (Pilots) Core leads are engaged throughout the entire process, particularly during the letter of intent (LOI) and application review stages. The CTSI Executive Committee hosted a translational science educational session for potential pilot applicants and the Penn State community in July 2023, and a Translational Research vs. Translational Science presentation in August 2024. Individual Core leads and staff support the Core consultation process and the team science collaboration planning sessions.

### Existing supports

Pilot awardees granted before 2023 were offered access to existing CTSI services during their project period. This was done passively by directing pilot project PIs and team members to the list of available support services on the CTSI website, and by connecting pilot project PIs with the appropriate CTSI service when assistance was requested or when a barrier to project success was mentioned in a progress report. These existing services include 1) recruitment and retention support, 2) access to the CTSI Research Navigator (added in 2023), and 3) consultations with CTSI Cores.

Recruitment and retention support is provided through in-person consultation with CTSI specialists, who provide direction on inclusive approaches to recruitment (e.g. utilization of Penn State’s LION Mobile Clinic to reach rural populations; partnerships with community-based organizations to broaden community reach). Additionally, the CTSI Recruitment Toolkit is disseminated during the consultation process to provide evidence-based practices for recruiting and retaining research participants, as well as to offer recruitment tools and templates for teams to tailor to their specific project needs [8]. Resources on how to effectively conduct community-engaged research, develop a recruitment plan, and recruit in rural populations are also described. The toolkit provides Penn State-specific guidance on how to get started in recruitment efforts. While this toolkit is available to all Penn State faculty, it is explicitly shared with pilot awardees when they indicate difficulty recruiting and retaining research participants in their interim progress report.

The second existing support mechanism is CTSI Core consultations. Consultations are available with all CTSI Cores, including Biostatistics, Epidemiology, and Research Design (BERD), Implementation Science Core (ISC), and Community Engaged Research Core (CERC). Additional details about Core consultations are provided under *New Supports in 2023* below. Consultations are available to all institutional faculty and staff, however, have been newly emphasized to pilot applicants. Although the content of the Core consultations did not change, the way we promoted and encouraged their use for pilot applicants was enhanced as part of this initiative.

The third existing support mechanism is the CTSI Research Navigator. Established in 2023, the Research Navigator is a clinical research professional with research project management experience and expertise as well as a working knowledge of both CTSI and Penn State-wide institutional research resources. The CTSI leverages a REDCap intake form for research inquiries from faculty and staff where individuals can select areas of need. In addition to being listed as a potential connection, the Research Navigator is automatically engaged if individuals select more than three resources from the list. This customer-service-focused approach supports staff and faculty through a one-on-one consultation to better understand their research requirements and how the CTSI and institution can support these efforts. Like the CTSI Core consultations, this resource was available to faculty prior to this initiative launch; however, contact with the Research Navigator was further promoted through this initiative. Pilot awardees are now offered consultation with the Research Navigator at the time of award to explore resources, which raises awareness of available research support.

### New supports in 2023

The first newly implemented support mechanism was a revised information session for potential pilot program applicants. This presentation provided an overview of the CTSI, the goals of the pilot funding program, the phases of translational research, and the difference between translational research and translational science. The presentation was recorded and uploaded to the CTSI YouTube playlist for those who could not attend synchronously. It has since been viewed over 240 times since originally presented. Additional topics covered during the session included funding eligibility, the application process, an explanation of the Core consultation process, budget guidelines, review criteria, and award conditions.

The second support mechanism implemented was a CTSI Core consultation phase, which was added to the application development timeline. An LOI down selection process was conducted by CTSI leadership, during which selected proposals were reviewed for opportunities to strengthen clinical and translational science. Cores were recommended for consultation with study teams to strengthen their proposal and improve their study design. These consultations were voluntary and at the discretion of the pilot project PI. However, the pilot project PIs could highlight how the project evolved through consultative feedback in their full proposal. If funded, applicants were encouraged to continue to seek assistance from CTSI Cores throughout the project period to expand their team’s expertise and continually strengthen their research.

The third support mechanism added to the 2023 funding cycle was an orientation meeting to educate and assist teams on the internal and NCATS regulatory processes required to gain access to funds and expedite their project start. This meeting was modeled after the orientation meeting of the Institute of Clinical and Translational Sciences at Washington University in St Louis, which was shared at the NCATS CTSA Program Quality Assurance/Quality Control group [9]. The goal of our orientation meeting was to bring together pilot awardees to congratulate them on their award and provide an opportunity to share their research project with other awardees. This meeting allowed the Director to cultivate an ongoing collaborative relationship with the project leads. The agenda included a review of the timeline and process for submitting progress reports and education for pilot project PIs on the required internal and external regulatory and financial processes. The Director discussed the additional CTSI resources available to the teams for use throughout their project.

The fourth newly implemented support mechanism encouraged funded teams to participate in a 90-minute virtual collaboration planning session facilitated by our CTSI Team Science lead. Collaboration planning is an evidence-based team intervention that helps teams establish a roadmap for handling potential challenges before they happen by clarifying expectations and establishing operating procedures [11,12]. The University of Wisconsin-Madison Institute for Clinical and Translational Research developed the structure and content of the collaboration planning session and trained CTSA personnel across many institutions to facilitate the sessions [13]. The topics discussed include team goals, roles and responsibilities, team outputs, team culture, processes, project management, and collaboration planning maintenance. The outcome of collaborative planning is a written document specifying how the interdisciplinary teams will collaborate on the project, which pilot teams were required to turn in one month after the collaboration planning session. To evaluate effectiveness, participants completed a 12-question pre-survey prior to the session to assess the understanding of roles, responsibilities, and expectations among team members, as well as share their goals for participating in the session. A post-session survey assessed the same questions and also asked about one action the team member plans to take as a result of participation. To continue to provide team science support following the collaboration planning session, members were made aware of the CTSI Team Science Toolbox, which offers evidence-based techniques and interventions across the team life cycle (e.g., team formation, team launch, team maturation) [14].

### New support in 2024

At Penn State, there is a need to educate faculty, students, and staff on the difference between the domains of translational research and translational science. To fill this knowledge gap, we hosted a Translational Science Seminar session on this topic, highlighting the NCATS definitions of translation, translational research, and translational science through a panel discussion of translational research and translational science across the spectrum [15]. To supplement the translational science educational opportunity, the CTSI Core consultations were expanded to include a translational science consultation. The goal of this consultation is to assist the pilot project PI and team in crafting their translational science project to align with the NCATS definition and CTSI priority areas [16].

## Results

**Figure 2.**
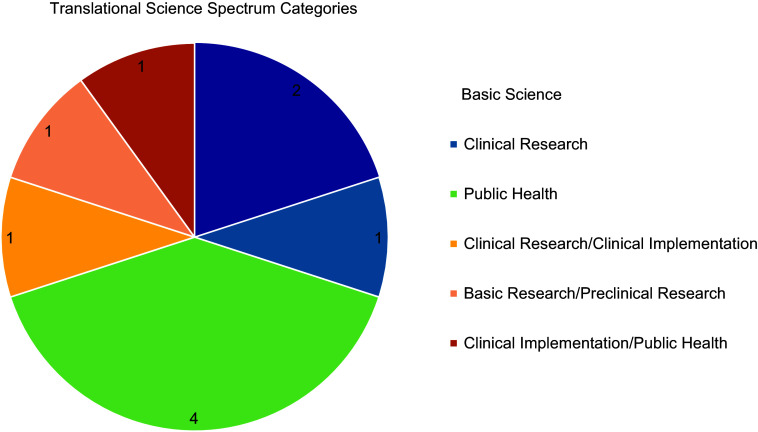
Translational science spectrum categories exhibited by proposals funded across 2023 and 2024 pilot funding cycles. Dark blue = basic science; light blue = clinical research; dark green = public health; yellow = clinical research/clinical implementation; light orange = basic research/preclinical research; dark orange = clinical implementation/public health.

In the 2023 and 2024 pilot funding cycles, we received a total of 55 LOIs, representing eight colleges across three Penn State campuses. Twenty-six projects were selected to move forward to the full application phase, of which 23 submitted a full application. A total of ten projects were awarded a pilot grant, representing two campuses, four colleges, and ten unique departments. The funded projects across the two cycles encompassed multiple different research methodologies, with investigators outlining plans to execute aims that included basic science research, prospective and retrospective observational analyses, and community-engaged evaluation and implementation. Funded proposals were classified according to the five categories of the Translational Science Spectrum, or a combination thereof, with Figure 2 depicting the domains represented across all funded proposals. Most projects funded across the two pilot funding cycles fell under the Public Health domain, along with multiple projects detailing aims that could be categorized as a combination of stages on the Translational Science Spectrum.

A timeline for the initiative is presented in Table 1.

**Table 1.** 2023 and 2024 pilot funding and initiative timeline

	Funding Cycle	
	*July 1, 2023 - June 30, 2024*	*July 1, 2024 - June 30, 2025*
Summer	Funding announcement released (July 1) Seminar: Pilot award information session (mid-July)	
		Seminar: Translational research vs. translational science seminar (August)
Fall	32 LOIs received (mid-September) CTSI Core consultations for 16 applicants (September – December)	23 LOIs received (mid-September) CTSI Core consultations for 26 applicants (September – December)
Winter	14 full applications received (mid-December)	9 full applications received (mid-December)
	Application review process (December – March)	
Spring	Applicants notified of funding decisions (April 1) 6 projects funded	Applicants notified of funding decisions (April 1) 4 projects funded
	Orientation meeting and collaboration planning sessions (May – June)	
Summer	Projects begin (July 1)	

**Figure 3.**
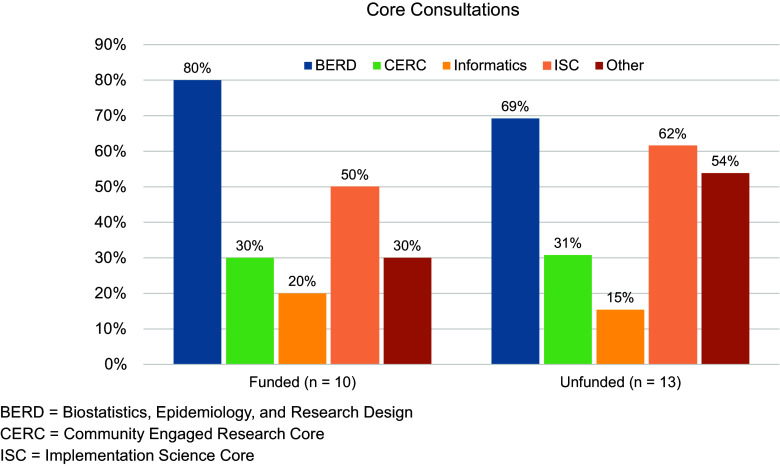
Percentage of teams who engaged in consultations with each CTSI core area by funded and unfunded proposals. Blue = BERD; green = CERC; yellow = informatics; light orange = ISC; dark orange = other.

The average number of faculty on the research team for both funded and unfunded applications was 3.3 (range = 1–6). Across 23 applications, the average overall priority score (using the NIH scoring criteria 1–9) was 3.9 (range = 2.7–7.8); the average overall priority score for funded applications was 3.3 (range = 2.7–4.3). Twenty-two of 23 pilot project PIs that submitted a full application participated in the CTSI Core consultations process. The average number of consultations suggested to each team was 2.4 (range = 1–3), and on average, teams engaged in 2.2 consultations (range = 0–4; average for funded teams = 2.1; average for unfunded teams = 2.3). The percentage of funded and unfunded teams who participated in Core consultations by CTSI Core area is presented in Figure 3.

The CTSI Cores and support areas represented in these completed consultations included BERD, ISC, CERC, Informatics, and others areas of Penn State strength. These CTSI Core consultations resulted in significant changes to the project proposals, providing suggestions for revised statistical methods and sample size calculations, as shared by project PIs: “*Consultation with the CTSI [BERD] core helped to accurately determine sample size to detect clinically meaningful changes in our proposed outcomes and to generate reproducible findings and FAIR data;”* additions of scientific frameworks for which to focus the project methodology: *“From our consultation with [ISC], we changed our implementation outcomes from the Consolidated Framework for Implementation Research and RE-AIM frameworks to Proctor’s Implementations Outcomes;”* and increased engagement with community partners: *“The development of the survey instrument will now incorporate feedback from Community Engagement Studio participant[s] after reviewing the impact of [CERC].”* Additional feedback regarding consultation with the ISC stated, *“This partnership is pivotal in translating our laboratory findings into tangible benefits for rural communities, which are often disadvantaged in accessing advanced medical care.”* Pilot project PIs shared their high value of this process. As stated by one of the applicants, *“The unique requirement to meet with CTSI Core Consultations was not only critical in helping our team develop a relationship for the funding period, but it has also provided a natural collaboration with CTSI to strengthen this work’s appeal beyond the proposed funding period.”*

Of the 2023 awardees, each of the six funded teams was represented at the orientation meeting in April 2024. Participants of the meeting reported the session was very helpful for them to know what was required of them during the just-in-time period of the award, including requirements for the NCATS prior approval process. Additionally, four teams completed the team science collaboration planning session prior to the project start date of July 1, 2024. Session participants reported that collaboration planning was a valuable experience (4.4/5.0, with 5.0 being “strongly agree”), the facilitator was effective in guiding the session (4.7/5.0), and that they would recommend collaboration planning to a colleague (4.4/5.0). When asked about one action they will take as a result of session participation, one participant said *“I think just participating in this series and coming up with a collaboration plan will, in and of itself, improve the communication and collaborative nature of our team dynamics”* A six-month follow-up survey was used to evaluate the long-term outcomes of the training. Of the 11 survey respondents, eight agreed or strongly agreed that the training enhanced the team’s ability to collaborate effectively.

Pilot project awardees from previous cohorts who had received support through the existing support mechanisms such as the Research Navigator have also positively benefited from the supports offered. As stated by a previous researcher supported by the Research Navigator, *“I absolutely LOVED [the Research Navigator]!! I would not change that for anything! I totally depended on her for so much throughout the entire process. I could not have done this without her. Everyone should have a 1:1 with [the Research Navigator]!”* Current pilot project PIs were personally connected with the Research Navigator as part of their just-in-time process.

## Discussion

The goal of this initiative is to provide support to pilot project applicants and awardees throughout the entire funding cycle, in part by connecting them to new and existing CTSI Cores and other resources and services. Prior work has demonstrated that CTSA services are often underused, with users only engaging with one Core area as opposed to having cross-Core interactions [4]. These Core services act as key facilitators for research activities and are an opportunity to enhance research projects, enabling pilot teams to carry out the objectives of their pilot project and prepare for submissions of grant proposals, publications, and other dissemination efforts of their research findings [16]. Despite the recognition of CTSA service benefits, there has been little research that focuses on evaluating and refining pilot project support infrastructure. As such, our initiative seeks to overcome the silo effect that traditionally lessens an organization’s ability to cross-promote other resources that benefit investigators. This comprehensive centralization of services is designed to leverage the expertise of CTSI personnel and provide overarching support to investigators, allowing them to have a successful pilot award and future research endeavors.

### Future plans

We will continue to educate faculty, students, and staff on the difference between translational research and translational science. This will be done via virtual seminars as well as asynchronous learning modules using case studies focusing on each of the NCATS Translational Science Principles [16]. Pilot project PIs will also gain access to staff support through the CTSI Accelerated Staff Assistance Program (ASAP). This program provides research faculty with short-term or partial effort staffing support for human subjects’ research projects, thereby addressing the challenge of onboarding new staff to support projects with short timelines or filling part-time positions.

Additional plans for this initiative will focus on evaluation, including further developing the evaluation process for each of the identified support mechanisms. Continuous, comprehensive evaluation of the initiative using innovative approaches will allow us to monitor in real time the impact of the pilot projects we are supporting in relation to the Translational Science Benefits Model [7]. Further, we will be able to explore the initiative’s effect on pilot project success by comparing the current cohort of awardees to prior cohorts without comprehensive support. Using this model, we will be able to fully support pilot applicants from pre-application through follow-on funding. Finally, we will share best practices with other interdisciplinary research institutes at SPenn State and other CTSAs across the country. The opportunity for cross-university and cross-CTSA collaboration may provide additional ideas for support mechanisms for pilot projects PIs and their teams. By sharing the initiative framework and evaluation models, we hope to evaluate the outcomes and success of internally funded pilot projects across CTSA hubs, thus allowing us to identify areas of success and opportunities across the CTSA program.
